# Pulmonary artery intimal sarcoma with pleural effusion: a case report

**DOI:** 10.3389/fmed.2026.1783394

**Published:** 2026-04-13

**Authors:** Xian-Ying Xie, Xue-Jiao Yang, E. Guo, Yu-Lan Zheng

**Affiliations:** 1 School of Medicine, Wuhan University of Science and Technology, Wuhan, Hubei, China; 2 Department of Respiratory and Critical Care Medicine, Xiangyang Central Hospital, Affiliated Hospital of Hubei University of Arts and Science, Xiangyang, China

**Keywords:** case report, differential diagnosis, endovascular biopsy, pleural effusion, pulmonary artery intimal sarcoma

## Abstract

**Background:**

Pulmonary artery intimal sarcoma (PAIS) is a rare and aggressive malignancy that is frequently misdiagnosed as pulmonary thromboembolic disease because of overlapping clinical and imaging features.

**Case presentation:**

A 52-year-old woman was admitted in September 2025 due to progressive dyspnea, pleural effusion, and elevated D-dimer levels. Imaging analysis suggested potential pulmonary embolism, and thoracoscopic pleural biopsy and endovascular sampling revealed no evidence of malignancy. The persistent obstruction of the pulmonary artery, the absence of an identifiable thromboembolic source, and atypical angiographic findings raised the suspicion of an underlying malignant process. A repeat pulmonary angiography, accompanied by targeted endovascular biopsy, identified a spindle cell tumor with myxoid degeneration. Immunohistochemical and molecular analyses corroborated the diagnosis of PAIS. She experienced temporary symptomatic improvement following interventional treatment and subsequently underwent surgical pulmonary artery tumor endarterectomy. The postoperative course was uneventful.

**Conclusion:**

This case highlights the diagnostic challenges associated with PAIS and underscores the importance of considering PAIS in the differential diagnosis of pulmonary thromboembolic disease, particularly in patients with atypical pulmonary artery lesions and inconclusive initial investigations.

## Introduction

Pulmonary artery intimal sarcoma (PAIS) is a rare and highly aggressive malignant neoplasm originating from the intimal layer of the pulmonary artery (1). The estimated incidence of PAIS ranges from 0.001% to 0.03%, with only a limited number of cases documented globally (2). This condition predominantly affects adults across a wide age spectrum and is associated with a dismal prognosis, particularly in patients who are unable to undergo complete surgical resection, resulting in markedly reduced survival rates. The diagnosis of PAIS poses significant challenges, as its non-specific clinical manifestations often coincide with those of pulmonary embolism (2, 3). Definitive diagnosis necessitates histopathological confirmation, which can be difficult to achieve due to the limited accessibility of tissue samples. In this report, we present a rare case of PAIS that initially manifested as unilateral pleural effusion and pleural thickening. The patient underwent a comprehensive diagnostic evaluation, during which multiple imaging studies and biopsy attempts yielded non-diagnostic results. The condition was initially misdiagnosed as pulmonary embolism, thereby underscoring the diagnostic challenges associated with PAIS.

## Case presentation

**FIGURE 1:**
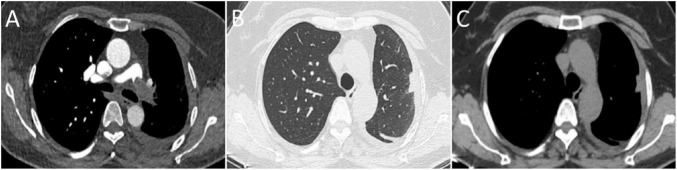
Computed tomography (CT) findings. **(A)** Computed tomography pulmonary angiography (CTPA) showing a filling defect in the left main pulmonary artery. **(B,C)** Non-contrast chest CT shows interstitial thickening at the peripheral region of the left upper lobe and focal nodular thickening of the left pleura.

**FIGURE 2:**
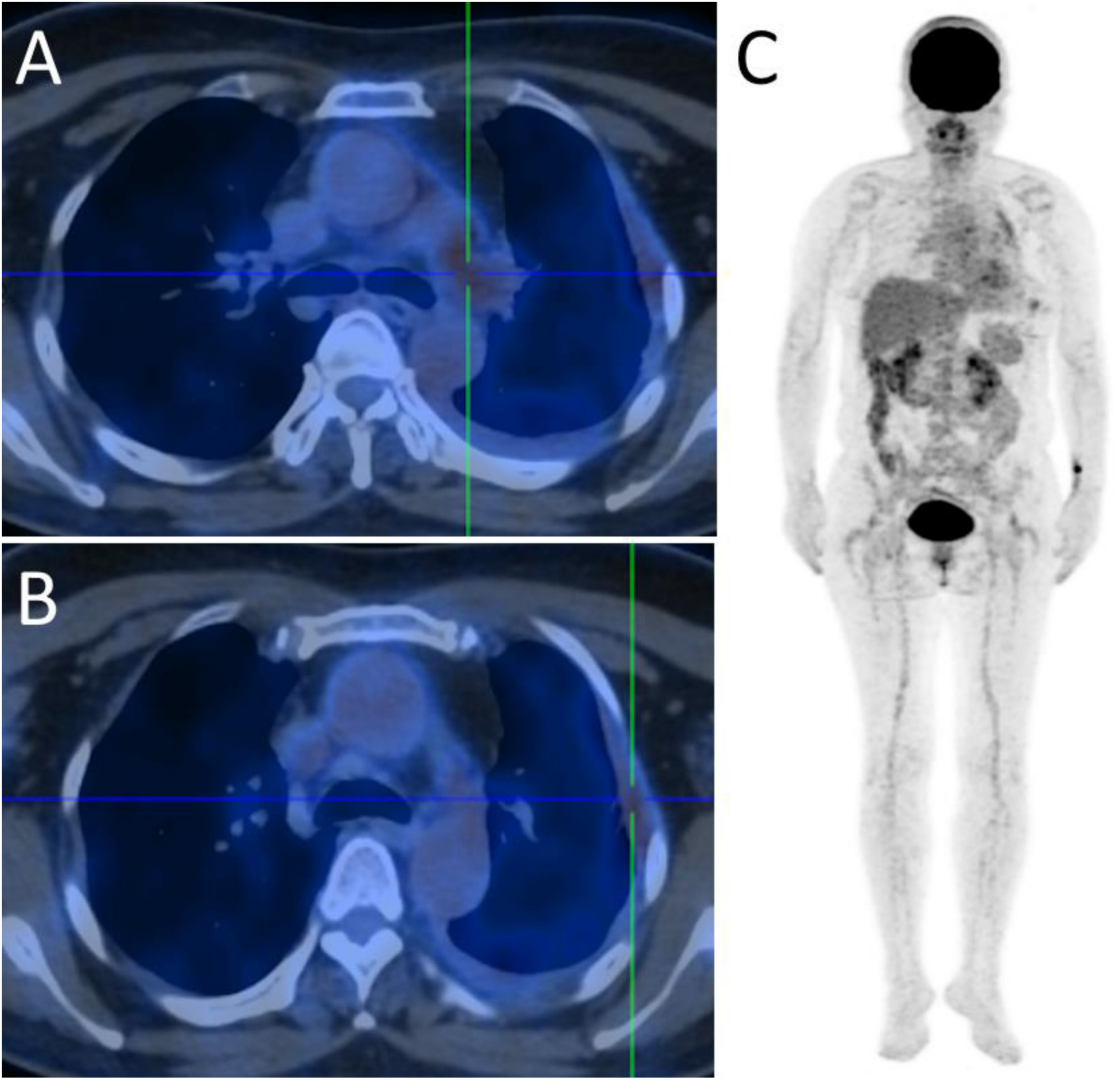
^18^F-fluorodeoxyglucose positron emission tomography-computed tomography (FDG PET-CT) images. **(A)** FDG PET-CT demonstrating mildly increased FDG uptake in the left pulmonary artery, with a SUVmax of 3.9 on early imaging and 5.0 on delayed imaging, involving an area measuring approximately 1.5 × 1.0 cm. Corresponding CT images show a patchy area of slightly decreased attenuation at the same site. **(B)** Mildly increased FDG uptake is also observed in the left pleura, which appears irregularly thickened, with a SUVmax of 3.7 on early imaging and 3.5 on delayed imaging. Additionally, patchy subpleural high-density opacities are noted in the left upper and lower lobes, the largest measuring approximately 2.2 × 1.0 cm, with mildly increased FDG uptake (SUVmax 3.0). **(C)** Whole-body PET-CT image showing the absence of abnormal hypermetabolic uptake elsewhere in the body.

In September 2025, a 52-year-old female patient was admitted to Xiangyang Central Hospital with a 2-day history of intermittent cough and sputum production, accompanied by progressive dyspnea that had persisted for approximately 2 weeks. Her medical and familial histories were unremarkable. In June 2025, she reported experiencing 10 days of exertional chest tightness, during which transthoracic echocardiography revealed no significant abnormalities. Coronary angiography demonstrated mild, non-obstructive coronary artery disease, characterized by 30% stenosis in the proximal to mid left anterior descending artery and 40% stenosis in the mid left circumflex artery, while coronary flow was preserved (TIMI grade 3). Laboratory tests indicated an elevated D-dimer level (1.12 mg/L FEU). Pleural fluid analysis revealed an exudative effusion (5,365 × 10^6^/L), predominantly lymphocytes (80%), lactate dehydrogenase (LDH, 528 U/L), and adenosine deaminase (ADA, 10 U/L). A repeat echocardiography demonstrated left atrial enlargement, with a left atrial diameter of 36 mm. Chest CT revealed left-sided pleural effusion, interstitial thickening at the periphery of the left upper lobe, and focal nodular thickening of the left pleura. Ambulatory electrocardiographic monitoring showed sinus rhythm with occasional atrial premature beats, short runs of atrial tachycardia, and non-specific anterior T-wave changes. Computed tomography pulmonary angiography (CTPA) showed extensive filling defects in the left pulmonary artery and its branches (Figure 1), suggestive of pulmonary embolism. ^18^F-fluorodeoxyglucose positron emission tomography-CT (FDG PET-CT) revealed mildly increased metabolic activity in the left pulmonary artery and subpleural regions, with a maximum standardized uptake value (SUVmax) of 5 (Figures 2A, B). Whole-body PET-CT imaging did not demonstrate any additional areas of abnormal hypermetabolic uptake elsewhere in the body (Figure 2C). Video-assisted thoracoscopic pleural biopsy showed no evidence of malignancy. Tuberculosis polymerase chain reaction testing was negative, rheumatologic and immunologic evaluations were unremarkable, and Doppler ultrasonography of the lower extremities revealed no evidence of deep vein thrombosis. She then underwent right heart catheterization, pulmonary angiography, catheter-directed thrombectomy, and pulmonary artery biopsy. Histopathological examination showed no evidence of malignancy. Immunohistochemical staining demonstrated negativity for cytokeratin (CK), focal positivity for desmin and smooth muscle actin (SMA), negativity for S-100 protein, focal positivity for MDM2, positivity for CD34, and a Ki-67 labeling index (LI) of approximately 1%. The persistence of pulmonary artery obstruction raised concern for an underlying structural lesion.

**FIGURE 3:**
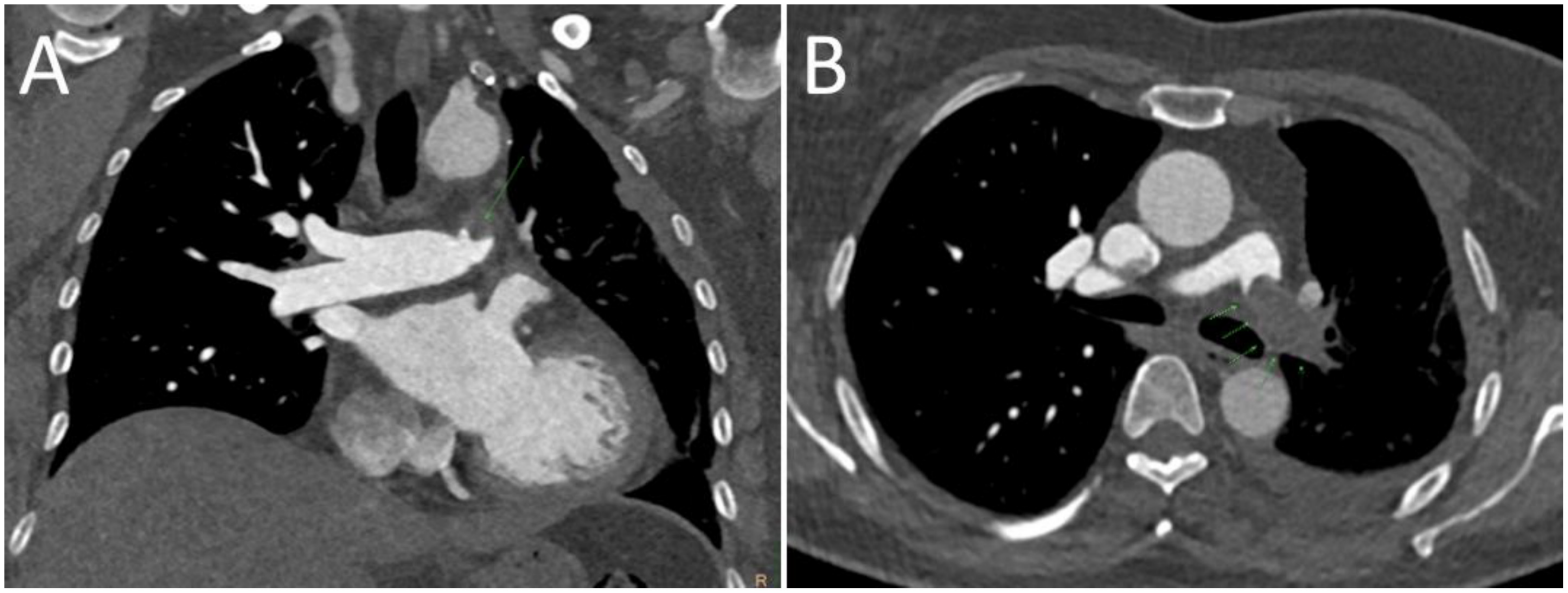
Computed tomography pulmonary angiography (CTPA) of the left pulmonary artery. **(A)** Coronal view. **(B)** Axial view. Green arrows indicate a low-attenuation intraluminal filling defect within the left main pulmonary artery.

**FIGURE 4:**
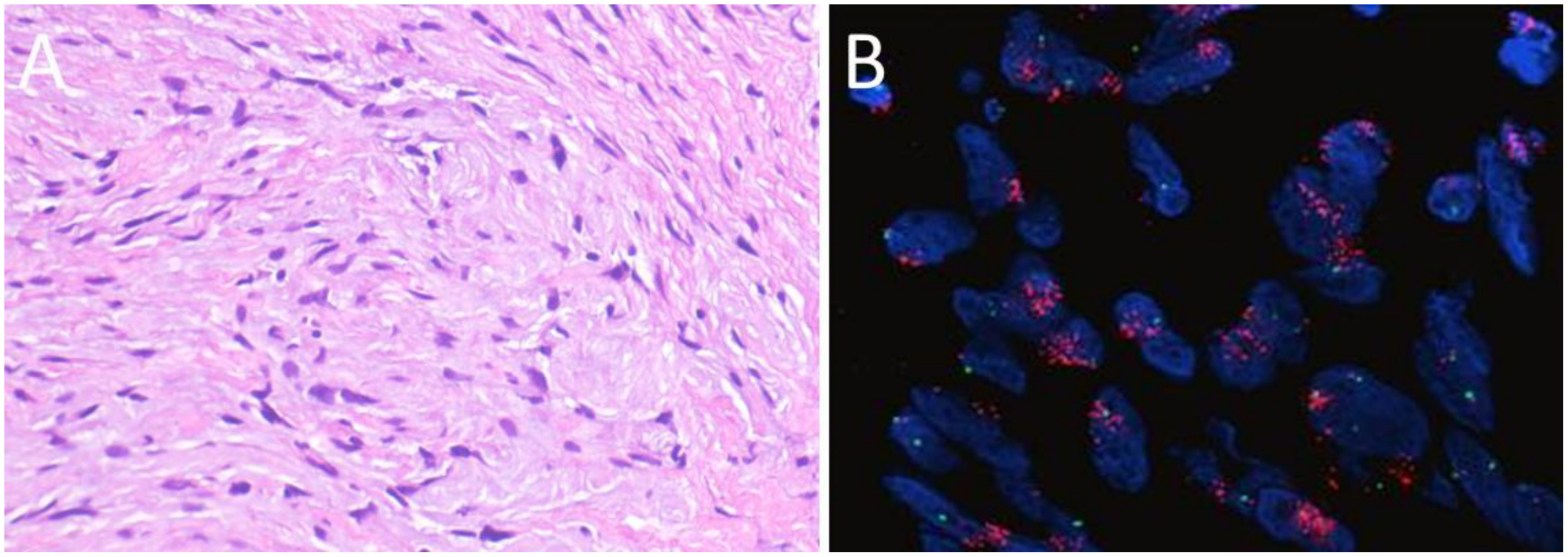
**(A)** Histopathological findings show predominantly proliferative spindle cells arranged in a relatively loose pattern, exhibiting fibroblast-like morphology. Occasional cells with enlarged, hyperchromatic nuclei and prominent nucleoli are observed, without marked cytologic atypia. Scant inflammatory cell infiltration is present within the stroma (H&E staining, original magnification × 400). **(B)** Fluorescent *in situ* hybridization (FISH) showing MDM2 amplification.

**FIGURE 5:**
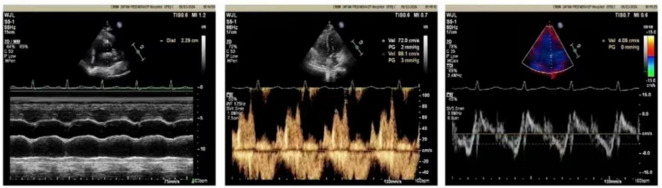
Follow-up echocardiographic images, demonstrating normal cardiac chamber size and preserved ventricular systolic function, with no significant valvular abnormalities.

A repeat pulmonary angiography with thrombectomy and biopsy was conducted in November 2025. CTPA revealed complete occlusion of the left main pulmonary artery, accompanied by an intraluminal mass (Figure 3). Cytological examination of the pulmonary arterial blood yielded negative for malignant cells. However, histological analysis of the pulmonary artery biopsy identified a spindle cell tumor (Figure 4A). Immunohistochemical analysis demonstrated positivity for vimentin, CD31, CD34, STAT6, and MDM2, while showing negativity for pancytokeratin (PCK), leukocyte common antigen (LCA), SMA, S-100 protein, Sox-10, CD68, and WT-1. Fluorescent *in situ* hybridization (FISH) also showed MDM2 amplification (Figure 4B). The Ki-67 LI was approximately 10%, supporting a diagnosis of PAIS with myxoid degeneration. She subsequently underwent femoral venous access with catheterization, pulmonary artery angioplasty, super-selective pulmonary angiography, and a repeat pulmonary artery biopsy. This procedure resulted in a significant improvement in dyspnea, and no major perioperative complications were observed. In December 2025, she underwent surgical pulmonary artery tumor endarterectomy, and the postoperative course was uneventful. Follow-up chest CT demonstrated a reduction in left-sided pleural effusion compared with prior imaging. The previously noted interlobular septal thickening in the left lower lobe had partially improved, and subpleural thickening showed interval absorption. No evidence of recurrence was observed on follow-up imaging. Follow-up echocardiography demonstrated normal cardiac chamber size and preserved ventricular systolic function, with no significant valvular abnormalities (Figure 5).

## Discussion

Pulmonary artery intimal sarcoma is an exceptionally rare and aggressive malignancy that poses substantial diagnostic challenges in clinical practice. In the early stage, its clinical manifestations are non-specific and frequently overlap with pulmonary embolism, which has a much higher incidence rate, often leading to delayed or incorrect diagnosis. In the present case, the patient experienced a prolonged and complex diagnostic trajectory. Initial imaging was dominated by pleural effusion rather than overt pulmonary vascular obstruction, while subsequent CTPA revealed intraluminal filling defects, corroborated by elevated D-dimer levels, which resulted in an initial diagnosis of pulmonary embolism. Although initial examinations did not indicate the presence of malignancy, the persistence of intraluminal obstruction observed on serial angiography, coupled with the absence of an identifiable thromboembolic source, sustained suspicion of an underlying malignancy. The definitive diagnosis of PAIS with myxoid degeneration was ultimately confirmed through repeated pulmonary angiography combined with targeted endovascular sampling. This case highlights the need to maintain suspicion for PAIS in patients with persistent pulmonary artery filling defects, inconclusive biopsy findings, and atypical clinical features, even when initial investigations favor pulmonary embolism. Notably, the mild left atrial enlargement observed in this case was considered a non-specific finding and was not thought to be directly related to the pulmonary artery lesion.

Several recent case reports have highlighted the diagnostic challenges of PAIS. Atahan et al. described a case initially misdiagnosed as pulmonary embolism, with definitive diagnosis established only after surgical resection and molecular confirmation of MDM2 amplification (4). Ioakeimidou et al. also reported a patient with recurrent pulmonary embolism-like episodes, who was ultimately diagnosed with PAIS (5). Yeh et al. emphasized the technical difficulty and limited yield of endovascular biopsy, often requiring repeated tissue sampling for diagnosis (6). Collectively, these reports underscore that PAIS is frequently misinterpreted as pulmonary thromboembolic disease and that obtaining adequate intravascular tissue for histopathological confirmation remains technically challenging. The present case further illustrates these diagnostic difficulties while adding additional complexity. Unlike the more typical presentation dominated by pulmonary arterial mass or recurrent embolic episodes, our patient initially presented with unilateral pleural effusion and pleural thickening, further obscuring early suspicion of malignancy. Moreover, the moderate FDG uptake on PET-CT and multiple non-diagnostic biopsy attempts prolonged the diagnostic process. Definitive diagnosis was achieved only after repeated endovascular sampling integrated with histopathologic and molecular evaluation. This case highlights the importance of recognizing atypical presentations and adopting a persistent, multimodal diagnostic approach when pulmonary artery obstruction remains unexplained.

Several clinical and radiological features may aid in differentiating PAIS from pulmonary thromboembolic disease. Firstly, pulmonary embolism typically shows symptomatic and radiological improvement after anticoagulation and is frequently associated with deep vein thrombosis (7), whereas PAIS is generally unresponsive to anticoagulant therapy and rarely accompanied by venous thrombosis. Secondly, imaging characteristics also provide important clues, as PAIS often appears on CT as a lobulated or polypoid intraluminal soft-tissue mass with heterogeneous enhancement, rather than organized thrombus (8). In our case, DSA demonstrated a focal intraluminal mass in the left pulmonary artery, favoring a neoplastic process. In addition, FDG PET-CT may support the differential diagnosis, as pulmonary artery sarcoma usually shows higher metabolic activity than pulmonary embolism. Ito et al. demonstrated that the mean SUVmax of pulmonary artery sarcoma was markedly higher than that of pulmonary embolism (9). Although FDG uptake in our patient was only moderately increased, it nevertheless raised suspicion for an underlying malignancy.

Pathologically, pulmonary artery intimal sarcoma is characterized by an intravascular growth pattern accompanied by marked morphologic and immunophenotypic heterogeneity. Macroscopically, PAIS typically presents as an intravascular mass closely adherent to the vascular intima, with potential longitudinal extension along the pulmonary arterial tree (10, 11). Histologically, the tumor is commonly composed of spindle or epithelioid cells with variable atypia, frequently accompanied by myxoid or fibrotic changes (12, 13), as observed in the present case. Immunohistochemically, PAIS demonstrates a largely non-specific profile, with variable expression of mesenchymal markers such as desmin and SMA, while lacking epithelial and lineage-specific markers, including CK/PCK, LCA, S-100 protein, and Sox-10 (8). While this immunoprofile serves to exclude epithelial, lymphoid, and neural tumors, it does not directly confirm PAIS. Consequently, immunohistochemistry alone may be insufficient for definitive diagnosis in small pulmonary artery biopsy specimens. Notably, FISH has shown frequent amplification of MDM2 (65%), PDGFRA (81%), and EGFR (76%) in PAIS (12), although both MDM2-positive and MDM2-negative cases have been documented (8, 13). Therefore, the final diagnosis of PAIS relies on an integrated assessment of the characteristic intraluminal growth pattern, spindle cell morphology with myxoid change, supportive molecular findings, and exclusion of other tumor entities. In the present case, MDM2 positivity identified on repeat pulmonary artery biopsy, in conjunction with characteristic morphologic features, was pivotal in establishing the final diagnosis. It is imperative to underscore that the acquisition of tissue from the pulmonary artery presents significant technical challenges and inherent procedural risks. Consequently, early biopsies may result in non-diagnostic outcomes due to superficial sampling or inadequate tissue volume, as demonstrated in this case, where the initial pulmonary artery biopsy returned negative results, while a subsequent biopsy successfully yielded diagnostic tissue. In instances where clinical suspicion persists, it is advisable to consider more aggressive and targeted endovascular strategies to secure sufficient deep intraluminal specimens.

Currently, there is no standardized treatment strategy for pulmonary artery intimal sarcoma; however, multimodal therapy and complete surgical resection appear to confer a survival benefit when feasible (3). In the present case, balloon angioplasty provided temporary symptomatic relief, necessitating further management, which requires multidisciplinary evaluation and close follow-up.

## Conclusion

Pulmonary artery intimal sarcoma is a rare yet aggressive malignancy that is frequently misdiagnosed as pulmonary embolism due to overlapping clinical manifestations, particularly progressive dyspnea. In patients with suspected pulmonary embolism who present with atypical features such as pleural effusion, poor response to conventional therapy, absence of an identifiable thromboembolic source, or increased metabolic activity of intraluminal pulmonary artery lesions on PET-CT, PAIS should be considered early in the differential diagnosis. Timely recognition and proactive acquisition of adequate intravascular tissue through interventional approaches are critical for establishing a definitive diagnosis.

## Data Availability

The original contributions presented in this study are included in this article/supplementary material, further inquiries can be directed to the corresponding author.

## References

[1] Mussot S Ghigna M Mercier O Fabre D Fadel E Le Cesne A et al. Retrospective institutional study of 31 patients treated for pulmonary artery sarcoma. *Eur J Cardio-Thoracic Surg.* (2013) 43:787–93. 10.1093/ejcts/ezs387 22843511

[2] Assi T Kattan J Rassy E Moussa T Nassereddine H Honore C et al. A comprehensive review on the diagnosis and management of intimal sarcoma of the pulmonary artery. *Crit Rev Oncol Hematol.* (2020) 147:102889. 10.1016/j.critrevonc.2020.102889 32035299

[3] Blackmon S Rice D Correa A Mehran R Putnam J Smythe W et al. Management of primary pulmonary artery sarcomas. *Annals Thoracic Surg.* (2009) 87:977–84. 10.1016/j.athoracsur.2008.08.018 19231448

[4] Atahan C Güral Z Yücel S Ağaoğlu F. Pulmonary artery intimal sarcoma: case report of a patient managed with multimodality treatment and a comprehensive literature review. *Strahlentherapie Onkol.* (2024) 200:725–9. 10.1007/s00066-024-02250-6 38866999 PMC11272804

[5] Ioakeimidou S Stephan F Pluess E Seeman M Srivastava D. Pulmonary artery intimal sarcoma mimics recurrent pulmonary artery embolism. *Eur J Case Reports Internal Med.* (2025) 12:005150. 10.12890/2025_005150 40636225 PMC12236683

[6] Yeh J Hsiao S Roan J Chen Y Liu P Chang H. Endovascular biopsy for pulmonary artery sarcoma mimicking pulmonary embolism. *Acta Cardiol Sinica.* (2023) 39:658–62. 10.6515/acs.202307_39(4).20230306c 37456939 PMC10346053

[7] Kim C Kim M Kang J Song J Lee K Kim S. Pulmonary artery intimal sarcoma versus pulmonary artery thromboembolism: Ct and clinical findings. *Korean J Radiol.* (2018) 19:792–802. 10.3348/kjr.2018.19.4.792 29962886 PMC6005959

[8] Li Y Li L Tong H Xu H Ma S Yang L et al. Pulmonary artery intimal sarcoma mimicking pulmonary thromboembolism: a case report. *Medicine.* (2021) 100:e24699. 10.1097/md.0000000000024699 33578605 PMC10545097

[9] Ito K Kubota K Morooka M Shida Y Hasuo K Endo H et al. Diagnostic usefulness of 18f-Fdg Pet/Ct in the differentiation of pulmonary artery sarcoma and pulmonary embolism. *Ann Nuclear Med.* (2009) 23:671–6. 10.1007/s12149-009-0292-y 19680740

[10] Burke A Virmani R. Sarcomas of the great vessels. A clinicopathologic study. *Cancer.* (1993) 71:1761–73. 10.1002/1097-0142(19930301)71:53.0.co;2-7 8448740

[11] Jo V Doyle L. Refinements in sarcoma classification in the current 2013 world health organization classification of tumours of soft tissue and bone. *Surg Oncol Clinics North Am.* (2016) 25:621–43. 10.1016/j.soc.2016.05.001 27591490

[12] Bode-Lesniewska B Zhao J Speel E Biraima A Turina M Komminoth P et al. Gains of 12q13-14 and overexpression of Mdm2 are frequent findings in intimal sarcomas of the pulmonary artery. *Virchows Arch Int J Pathol.* (2001) 438:57–65. 10.1007/s004280000313 11213836

[13] Cheah D Wong R Gupta S Bellafiore F Dakshinesh P. Obscure pulmonary artery intimal sarcoma presenting with hemoptysis and pulmonary embolism. *Curr Problems Cancer Case Rep.* (2025) 17:100351. 10.1016/j.cpccr.2025.100351

